# A real-time system for biomechanical analysis of human movement and muscle function

**DOI:** 10.1007/s11517-013-1076-z

**Published:** 2013-07-25

**Authors:** Antonie J. van den Bogert, Thomas Geijtenbeek, Oshri Even-Zohar, Frans Steenbrink, Elizabeth C. Hardin

**Affiliations:** 1 Department of Mechanical Engineering, Cleveland State University, 1960 E. 24th Street, SH 232, Cleveland, OH 44115 USA; 2 Orchard Kinetics LLC, 2217 S. Overlook Rd., Cleveland, OH 44106 USA; 3 Motek Medical B.V., Keienbergweg 77, 1101 GE Amsterdam, The Netherlands; 4 Cleveland VA Medical Center, 10701 East Boulevard, Cleveland, OH 44106 USA

**Keywords:** Gait, Movement analysis, Biomechanics, Real-time, Virtual reality

## Abstract

Mechanical analysis of movement plays an important role in clinical management of neurological and orthopedic conditions. There has been increasing interest in performing movement analysis in real-time, to provide immediate feedback to both therapist and patient. However, such work to date has been limited to single-joint kinematics and kinetics. Here we present a software system, named human body model (HBM), to compute joint kinematics and kinetics for a full body model with 44 degrees of freedom, in real-time, and to estimate length changes and forces in 300 muscle elements. HBM was used to analyze lower extremity function during gait in 12 able-bodied subjects. Processing speed exceeded 120 samples per second on standard PC hardware. Joint angles and moments were consistent within the group, and consistent with other studies in the literature. Estimated muscle force patterns were consistent among subjects and agreed qualitatively with electromyography, to the extent that can be expected from a biomechanical model. The real-time analysis was integrated into the D-Flow system for development of custom real-time feedback applications and into the gait real-time analysis interactive lab system for gait analysis and gait retraining.

**Electronic supplementary material:**

The online version of this article (doi:10.1007/s11517-013-1076-z) contains supplementary material, which is available to authorized users.

## Introduction

Biomechanical analysis of human movement has become an important tool for basic research and for clinical management of orthopedic and neurological conditions. Clinical movement analysis is traditionally performed off-line by processing of previously recorded raw motion and force data, resulting in a laboratory or gait report to the clinician who makes treatment decisions. Clinically relevant information in the report typically includes the time histories of biomechanical variables such as joint angles (kinematics) and joint moments (kinetics) [15]. In recent years, musculoskeletal models have been used to provide additional information about muscle length changes [2] and muscle forces [8, 9, 12, 30].

A real-time biomechanical analysis, as opposed to a report that is generated during post-processing, would create unique opportunities for both the patient and the therapist to interact in real-time with biomechanical data during patient examination or treatment. Clinicians and physical therapists could benefit from a real-time visualization and quantification of specific motion variables, as well as from having additional information about internal forces and moments which would remain otherwise fundamentally invisible. Furthermore, such biomechanical data can also be presented to the patient in real-time, to help them perform therapeutic exercises more effectively than could be done with verbal or tactile feedback from a physical therapist [10].

Custom applications have been developed for feedback training using specific variables computed in real-time, such as a single joint angle [3] or a single joint moment [25]. To make real-time computation feasible, approximations are often used that neglect certain mechanical effects, such as inertial terms in the equations of motion [25]. Real-time commercial systems are currently limited to kinematic variables (joint angles) [3, 27] and possibly joint moments, but do not include muscle variables. Although angles and moments can be a useful surrogate for tissue loads and muscle recruitment that are relevant to orthopedic or neurological rehabilitation, an analysis at the muscle level is needed for a full understanding [8, 9]. This is, however, computationally demanding because muscle forces must be estimated simultaneously for all muscles in a limb, or ideally, in the whole body [8, 9]. Consequently, currently available software systems for analysis of muscle function (Anybody, www.anybodytech.com; and OpenSim [8]) do not perform real-time analysis.

In this paper we present a full human body model (HBM) that can produce a real-time analysis of 3D kinematics, kinetics, and muscle function. The goals of this paper are (1) to present the model and the methods of computation, and (2) to present results from a group of able-bodied subjects.

## Methods

### Numerical methods

**Fig. 1:**
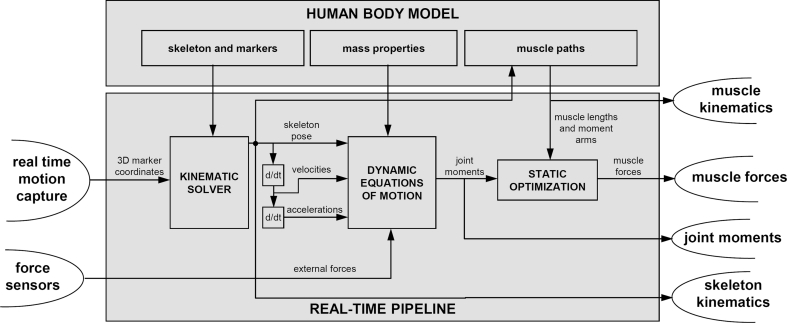
Data flow within the human body model (HBM)

Within the HBM, the processing pipeline consists of inverse kinematics, low-pass filtering, inverse dynamics, muscle kinematics (length change and moment arms), and muscle force estimation (Fig. 1). In order to keep up with an input stream of 120 frames per second (fps), which is typical for inverse dynamic analysis, the total computation time for all processing steps must be <8.33 ms per frame.

The kinematic model in HBM consists of 16 rigid body segments that are coupled by joints, with a total of 44 kinematic degrees of freedom. Subject-specific joint centers and axes are calculated from 3D coordinates of markers attached to anatomical landmarks, while the subject is in an initialization pose. Details can be found in “Supplemental Material”. Inertial properties for all body segments are estimated during initialization from segment lengths and total body mass using published regression equations [6]. Forward kinematic equations were generated to express the global 3D position $\vec{r}_{i} ({\mathbf{q}})$ of a marker *i* as a function of the 44 generalized coordinates **q**. Given a set of marker coordinates $\vec{r}_{{i,{\text{meas}}}}$ measured by the motion capture system, the inverse kinematic problem is to find the model pose **q** that best fits the marker data. This was formulated as a nonlinear least-squares problem:

1 $${\mathbf{q}} = \arg \mathop {\hbox{min} }\limits_{{\mathbf{q}}} \sum\limits_{i = 1}^{N} {\left\| {\vec{r}_{i} ({\mathbf{q}}) - \vec{r}_{{i,{\text{meas}}}} } \right\|^{2} } $$

A full body marker set consisting of *N* = 47 markers was defined (see “Supplemental Material”) to provide redundancy and robustness against occasional marker dropout which is inevitable in real-time motion capture. After solving (1), the estimated body pose is processed by a real-time low-pass filter (second order Butterworth) that outputs the smoothed pose **q** as well as the generalized velocities ${\dot{\mathbf{q}}}$ and generalized accelerations $\ddot{{\mathbf{q}}}$. Details on the filter and its implementation are presented elsewhere [29]. The user would set the cutoff frequency of the filter based on the bandwidth of the movement that is being studied. Force platform data were processed with the same filter to prevent impact artifacts in the subsequent inverse dynamic calculations [16].

In the inverse dynamics processing step, a vector ${\varvec{\tau}}$ of unknown forces and moments, associated with the kinematic degrees of freedom, is solved from the multibody equations of motion:

2 $${\varvec{\tau}} = {\mathbf{M}}({\mathbf{q}})\ddot{\mathbf{{q}}} + {\mathbf{c}}({\mathbf{q}},{\dot{\mathbf{q}}}) + {\mathbf{B}}({\mathbf{q}}){\varvec{\tau}}_{\text{ext}} $$

where **M** is a square mass matrix, and **c** are terms related to Coriolis and centrifugal effects and gravity. The final term represents measured external forces (force plate data). Joint power was calculated as the product of joint moment and angular velocity. Separate equations were used to compute the full 6-DOF intersegmental loads at the knee, and these loads were expressed in the reference frame of the shank.

A total of 300 muscles are presently included in the model, based on previously published musculoskeletal models: 43 muscle elements in each lower extremity [7], 102 in each arm [4], and 10 in the spine [17]. The coupling between muscles and skeleton was represented by polynomials that compute total muscle–tendon length *L* as a function of skeleton pose **q**:

3 $$L({\mathbf{q}}) = \sum\limits_{i = 1}^{{N_{\text{terms}} }} {c_{i} } \prod\limits_{j = 1}^{{N_{\text{DOF}} }} {q_{i}^{{E_{ij} }} } $$

The number of terms will depend on how much detail is required to represent the function $L({\mathbf{q}})$. Based on the principle of virtual work [1], the muscle moment arm *d*_*k*_ with respect to a joint angle *k* is computed analytically by partial differentiation:

4 $$d_{k} = - \frac{{\partial L({\mathbf{q}})}}{{dq_{k} }} = - \sum\limits_{i = 1}^{{N_{\text{terms}} }} {c_{i} E_{ik} \prod\limits_{{{\text{j}} = 1}}^{{N_{\text{DOF}} }} {q_{i}^{{E_{ij} - \delta_{kj} }} } } $$

where $\delta_{kj}$ is the Kronecker delta. Coefficients *c*_*i*_ and exponents *E*_*ij*_ were obtained by stepwise regression to fit the polynomial model to moment arms obtained from OpenSim [8] for a sufficiently large set of skeleton poses **q**. The stepwise regression added successively terms (up to a maximum order) to the polynomial until difference in moment arm between polynomial and Opensim result was reduced to <2 mm. The muscle shortening velocity was computed as the dot product of moment arms **d** and generalized velocities ${\dot{\mathbf{q}}}$:

5 $$v = - \frac{{{\text{d}}L({\mathbf{q}})}}{{{\text{d}}t}} = - \sum\limits_{k} {\frac{{\partial L({\mathbf{q}})}}{{\partial q_{k} }}\frac{{{\text{d}}q_{k} }}{{{\text{d}}t}} = {\mathbf{d}}^{\text{T}} {\dot{\mathbf{q}}}} . $$

The final processing step performed static optimization to simultaneously estimate the forces **F** in all muscle elements. The optimization problem is formulated as a quadratic programming problem [9, 30]:

6 $$\begin{array}{*{20}c} {{\mathbf{F}} = \arg \mathop {\hbox{min} }\limits_{{\mathbf{F}}} \sum\limits_{i = 1}^{{N_{\text{muscles}} }} {V_{i} \left( {\frac{{F_{i} }}{{F_{{{ \hbox{max} },i}} }}} \right)}^{2} } \hfill \\ {\quad \quad {\text{subject to }}\left\{ {\begin{array}{l} {{\mathbf{D}}({\mathbf{q}}){\mathbf{F}} = {\varvec{\tau}}} \\ {F_{i} \ge 0} \\ \end{array} } \right.} \hfill \\ \end{array} \, $$

where $F_{{{ \hbox{max} },i}}$ is the maximal force that muscle *i* can produce and *V*_*i*_ is the muscle volume, which was assumed to be proportional to the product of maximal force and fiber length. These muscle properties were taken from the original models [4, 7, 17]. Weighting of the optimization objective by muscle volume is required to make the solutions independent of the level of discretization of the muscular anatomy [14]. The matrix ${\mathbf{D}}({\mathbf{q}})$ contains the moment arms $d_{ij}$ of muscle *j* with respect to kinematic variable *i*, which are dependent on joint angles **q** and computed using (4). Power generation of each muscle is now easily calculated as the product of muscle force and shortening velocity (5).

### Implementation

The HBM was implemented as a software library with a C/C++ application programming interface (API), coded with specific emphasis on real-time computation. C code for the forward kinematic model in (1) was generated using Autolev (Online Dynamics, Sunnyvale, CA, USA). The nonlinear optimization problem in (1) was solved with the Levenberg–Marquardt algorithm [20], with a Jacobian matrix for the forward kinematic model that was generated by symbolical differentiation in Autolev. The solution of each frame was used as the initial guess for the next frame. Solver iterations were terminated after a specified computation time, to ensure real-time performance. Autolev also generated the C code to compute the joint moments using (2). The static optimization problem (6) was solved with a recurrent neural network [32], simulated numerically with the forward Euler method up to a specified computation time for each frame. The result of each frame was used as initial condition for the next frame.

**Fig. 2:**
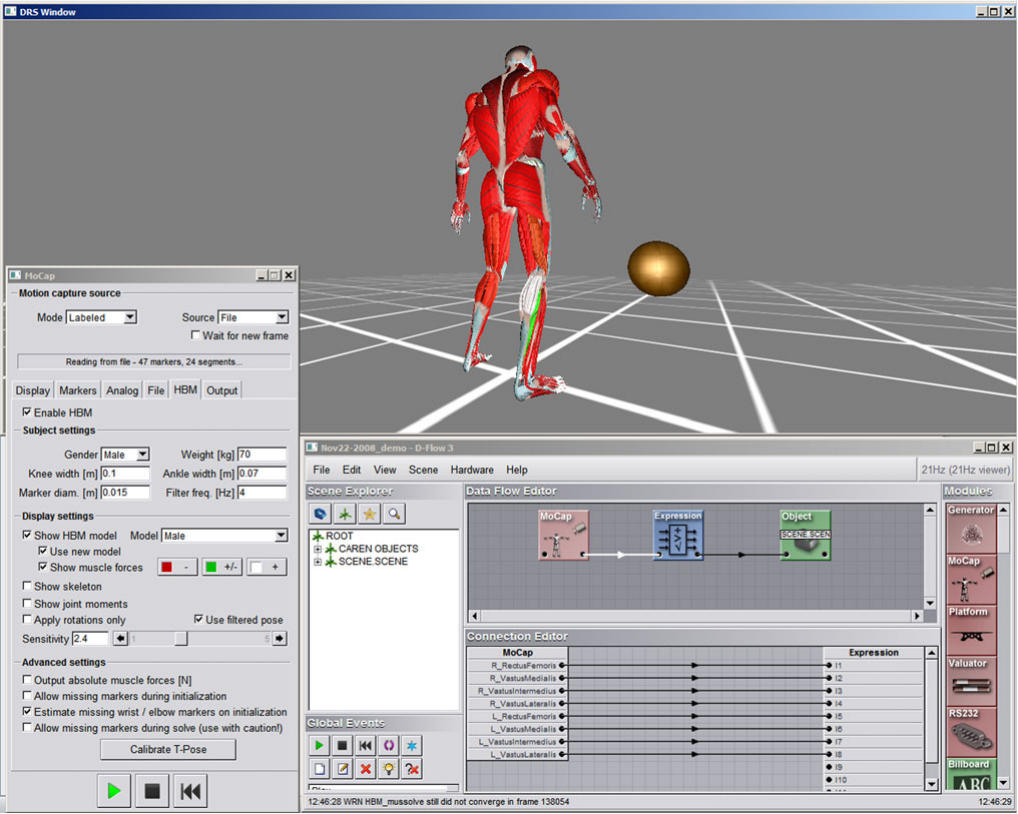
Screen image from the D-Flow system. The distributed rendering system (DRS) window is normally displayed on a large projection screen for interaction with patient and therapist. Muscle activation is visualized as a change in muscle color. The window on the *bottom right* is the console for application development, showing the data flow editor and the connection editor. A simple application is shown, in which estimated quadriceps forces are used to control a virtual ball, such that upward motion responds to total force, and horizontal motion responds to asymmetry. This simple application would help a patient train to increase their quadriceps activation while maintaining left–right symmetry. The window on the left is the user interface for the HBM

HBM was integrated in two applications. D-Flow (Motek Medical, Amsterdam, the Netherlands) provides a software development platform for custom applications that generate real-time feedback and visualization in a virtual reality environment [10]. Within D-Flow, biomechanical variables obtained from HBM can be visualized on an avatar using a coloring scheme to illustrate active muscles, or can used to control events and objects in a virtual environment providing many possibilities for rehabilitation, research and sports (Fig. 2). The lower extremity portion of HBM was also integrated in GRAIL (Gait Real-time Analysis Interactive Lab, Motek Medical, Amsterdam, the Netherlands) for clinical gait analysis and gait retraining. The results presented in this paper were obtained with HBM embedded in D-Flow version 3.10.1.

### Human subject data

Twelve healthy subjects (11 males and 1 female) volunteered to participate in this study which was approved by the Institutional Review Board of the Cleveland VA Medical Center. Average subject characteristics were: age 28.3 ± 3.9 years, body mass (with shoes) 75.9 ± 11.2 kg, and height 175 ± 8 cm. Subjects walked on a split-belt instrumented treadmill (ADAL3DM-F-COP-Mz, Tecmachine, France) for 30 s at their preferred walking speed and wearing their own shoes. Preferred walking speed was 0.97 ± 0.12 m/s with a gait cycle of 1.23 ± 0.09 s. During walking, kinematic marker data were collected at 100 Hz via a 16-camera passive marker motion capture system (Vicon, Oxford Metrics, UK) with the marker set described in “Supplementary Material”. Ground reaction forces were collected at 1,000 Hz from load cells in the treadmill.

For data processing, 100 frames were averaged from a standing trial for initialization of the subject-specific model. The low-pass filter was set to 6 Hz. Computation time limits for the iterative solvers were set to 1 ms for inverse kinematics, and 5 ms for static optimization. HBM was executed under Windows 7 on a 2.4 GHz Intel i5 CPU. All output variables were ensemble averaged over the 30-s trial to obtain one average gait cycle for each subject, from right heel strike to right heel strike. It was verified that the subjects had symmetrical gait, and therefore only the results from the right lower extremity will be presented.

On one subject, the analysis was performed at various computation time settings. Error due to premature termination of the iterative solvers was quantified as the overall root mean square (RMS) difference in joint angles and muscle forces between the test result and a result where there was no time limit for computation.

## Results

**Fig. 3:**
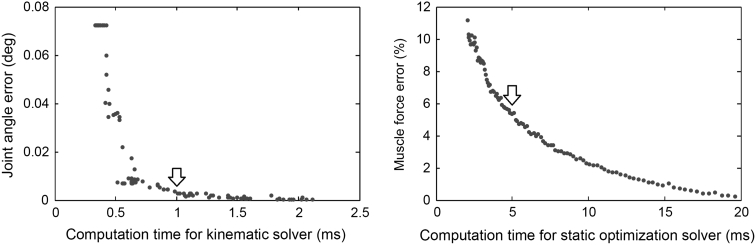
Errors in joint angles and muscle forces as a function of the allowed computation time in, respectively, the kinematic solver (*1*) and the static optimization (*6*). Results are presented for one representative subject. *Arrows* indicate the settings that are normally used for real-time analysis

With a computation time limit of 1 ms per frame, the kinematic solver (1) terminated, on average at 1.24 ms after doing four iterations. The low-pass filter required 0.07 ms, and the inverse dynamic calculation (2) required 0.41 ms. The iterative solver for the static optimization problem (6) performed, on average, 230 Euler integration steps in the allotted time of 5 ms. Errors due to time limits in the iterative solvers are shown in Fig. 3. At real-time speed settings, the errors due to premature termination of the iteration process were <0.01° for kinematics and <5 % for muscle forces. Figure 3 can be used to determine how these errors would change when the code is executed on faster or slower computer hardware, or when time limits are adjusted to a different frame rate for the streaming raw data.

**Fig. 4:**
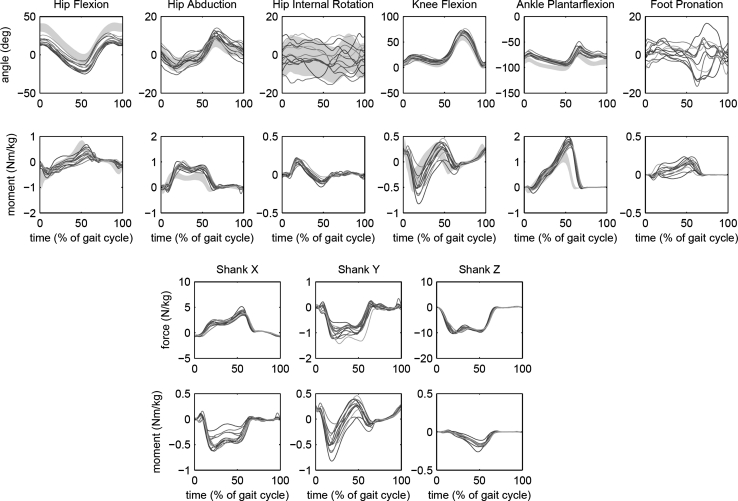
The *top* two *rows* show lower extremity joint angles and moments obtained with the human body model (HBM) from the 12 able-bodied subjects walking at preferred speed. Each *curve* represents one subject’s mean gait cycle. The *shaded area* represents mean and standard deviation from a study on children [24], for those variables that were available. Other joint-related variables are available in HBM, but not shown: joint angular velocity, and joint power generation. The *bottom* two *rows* show the inter-segmental loads at the knee, acting on the shank segment, and expressed using the *axes* of the shank reference frame: *X* (anterior), *Y* (lateral), and *Z* (superior)

Figure 4 (top panels) shows the lower extremity joint angles, moments, and powers obtained from all subjects. When available, results from the literature [24] were superimposed for comparison. Intersegmental knee loads are presented in the bottom panels of Fig. 4.

**Fig. 5:**
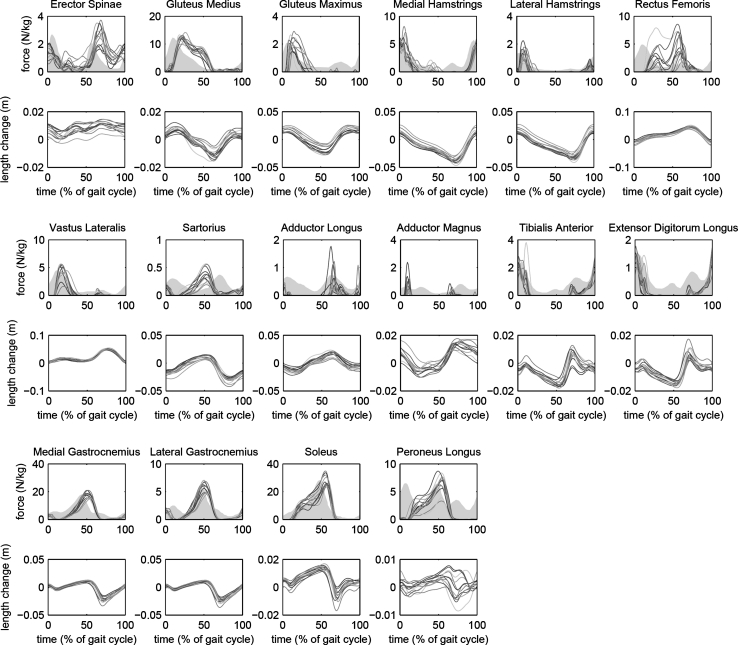
Forces and length changes for 16 muscle groups. EMG patterns from the literature [31] are shown for comparison, with the area under the EMG-time *curve* shaded. Amplitudes of the EMG patterns were scaled to coincide with the amplitude of estimated muscle force. Other muscle-related variables are available in HBM, but not shown: velocity of length change, power generation, and muscle activation (*F*/*F*_max_)

Muscle forces, length changes, shortening velocities, and powers in the lower extremity and spine are presented in Fig. 5 for 16 selected muscles, with electromyography (EMG) data from the literature [31] for visual comparison.

All results, including those not shown in figures, are available as “Supplementary Material”.

## Discussion

We have developed a system that performs a full biomechanical analysis of human movement in real-time. The analysis that is performed by the system is identical to existing approaches for inverse kinematic analysis [8], inverse dynamic analysis [30], and muscle force estimation [30]. The real-time performance is not achieved by simplifications of the model or the analysis, but by several innovations in computational methods to solve the analysis. Because the software does not need the capability to solve other models, the kinematic model and inverse dynamic model could be coded symbolically using the Autolev system. The resulting C code had a length of several megabytes, but was free from overhead due to loops, tests and branches, and function calls, and required only several milliseconds to execute. Muscle moment arm calculations were accelerated by using polynomials (3) that acted as lookup tables to produce results that were, for practical purposes, identical to the more time-consuming geometrical calculations performed by Opensim [8]. The static optimization problem to estimate muscle forces was solved by an iterative method [32] that eliminates the need to solve large systems of linear equations. It has been proved that this method produces the same solution as conventional methods for quadratic programming [32], when iterated long enough. In real-time applications, the initial guess is the result of the previous frame, and already very close to the correct solution. This allows us to terminate the iterations when the available computation time has been used up. Figure 3 shows that within 5 ms the solution is, on average, already within 5 % of the exact solution which would be reached when the algorithm is given unlimited computation time.

As configured, the total time to perform all model-based analyses was 6.72 ms, well within the requirement for real-time processing of streaming raw data at 120 fps, and a lag time that is sufficiently short for feedback and training applications. The kinematic analysis was hardly affected by allowing only 1 ms of computation, and could even be done at higher camera frame rates (when available) to maximize the benefit of noise reduction by low-pass filtering for estimation of velocities and accelerations. After the low-pass filtering, however, bandwidth is reduced and inverse dynamic analysis and static optimization can be performed at lower frame rate without loss of accuracy. This would reduce the load on the processor, or improve accuracy, or allow more complex models to be solved.

A low-pass filter was used to prevent noise in the inverse dynamic results, but unlike offline filtering, a time lag is inevitable in a real-time filter. The second order real-time Butterworth filter has a phase delay of 0.22/*f,* where *f* is the corner frequency [29]. With the 6 Hz filter that was used for the gait data, this amounts to 37 ms or about 4 % of the gait cycle. The results presented in Figs. 4 and 5 were not corrected for this delay; the results are presented as they would appear in a real-time application. This 4 % delay should be kept in mind when interpreting these results or comparing them to results from other studies.

Joint angles and moments (Fig. 4) showed the typical features that are usually seen in mechanical analysis of gait [24]. Differences between studies are inevitable because of study population and test protocol. Our results show lower knee and ankle moments (normalized to body mass) than [24] which is not surprising because of shoes and a higher length–mass ratio in adults. Hip moments are affected by the choice of reference frame [23]. We reported the joint moments in a joint coordinate system, rather than the thigh reference frame as in [24]. Other modeling assumptions have an affect as well, such as the definition of joint centers and joint axes. Details of the data processing can affect results. Our system, and Opensim [8], both use redundant marker sets to suppress to effect of soft tissue motion, while existing commercial systems for clinical movement analysis, such as used in [24], do not. The resulting differences can be substantial, but do not always interfere with clinical applications. The current practice is that each laboratory obtains their own normal reference data, using their study population, study protocol, and software system. The question may still be raised which system produces a more “correct” result, but this is outside of the scope of this paper.

Intersegmental forces and moments are useful for orthopedic questions related to joint injury. We have not yet implemented this for all joints in the model, but we do have this information available for the knee joint (Fig. 4), where these variables have been shown to be relevant to the risk of ACL injury [13] and progression of osteoarthritis [3, 25]. The ability to calculate knee joint loads and provide feedback on these variables in real time can help athletes and patients modify these variables via gait retraining exercises [3, 25]. Future versions of the software will provide information about intersegmental loads at all joints.

Estimated muscle forces (Fig. 5) had peaks that coincided with peaks in normal EMG [31] for most muscles, notable exceptions being the Sartorius and Rectus Femoris muscles. Similar relationships between muscle force and EMG are found in other modeling studies of walking [12, 28]. Perfect correlation can not be expected because EMG measures activation, not force. When there are major discrepancies in timing of peaks, however, it is likely that the force estimate is not correct. This can be caused by errors in the moment arms of the muscle in the model, or by the assumption that muscle force is distributed according to an optimization principle as stated in Eq. (6). These results show that users must be cautious when using the muscle force estimates, especially for certain muscles.

Analysis of muscle contraction kinematics and muscle forces is not yet well established in clinical movement analysis, but there are large potential benefits. For instance, information about muscle length change during gait can assist surgical planning for patients with cerebral palsy [2]. In stroke patients, estimation of muscle forces during gait can help identify specific deficits and compensatory strategies [19]. Software tools are already available for such analyses (Anybody and OpenSim) but these tend to be research-oriented and not sufficiently fast or user-friendly for clinical applications. Our system is, at this time, the only system that can perform muscle force estimation in real time. It is important that these estimates are validated before the system is applied clinically, and the validation must be done with a well-designed study that is relevant to the clinical question.

We performed the muscle force estimation using static optimization (6). This does not take into account the force–length or force–velocity properties, or internal dynamics of the muscles. Some of these properties are included in the OpenSim and Anybody systems, but this increases the computational cost but may not significantly improve the results in clinical applications [18]. The quadratic cost function [30] was chosen over the classical cubic cost function [5], mainly because it allowed us to use an efficient real-time solution method [32]. While the choice of cost function is subject of active research, the results of a static optimization seem to be rather robust with respect to the choice of cost function [11, 26]. A promising alternative is the minmax criterion [21], which would allow a real-time implementation but may lead to discontinuities in the muscle force trajectories [22]. A fundamental limitation of model-based muscle force estimation, as presented here, is that the same generic muscle models are used for all subjects. We assume standard anatomy (moment arms) and standard muscle strengths. Therefore, muscle force estimates may be biased towards normal in patients with neurological problems, muscle weakness, or pain. An approach to overcome such limitations was recently proposed [33], but this requires extensive patient calibration protocols which would be impractical in routine clinical use.

In conclusion, we have shown that a full biomechanical analysis of joint and muscle function can be obtained in real time, and that results are consistent between subjects and resemble previously published results. Real-time processing offers the unique opportunity for interactive use of biomechanical movement analysis in which the patient and therapist not only interact with each other, but also with biomechanical information that is presented to them in real time using advanced visualization methods (Fig. 2).

## Electronic supplementary material

Below is the link to the electronic supplementary material.

Detailed description of the model (PDF 672 kb)

Subject characteristics (XLS 24 kb)

Ground reaction force variables for each foot: 3D force (N/kg), center of pressure (m), free vertical moment (Nm/kg) (XLS 350 kb)

3D coordinates of the whole-body center of mass (m) (XLS 124 kb)

Kinematic analysis results (meters and degrees) (XLS 1212 kb)

Inverse dynamic analysis results (N/kg and Nm/kg) (XLS 1180 kb)

Joint power for each kinematic degree of freedom (W/kg) (XLS 1180 kb)

6-DOF intersegmental loads (N/kg and Nm/kg) (XLS 1208 kb)

Muscle forces (N/kg) (XLS 5803 kb)

Muscle activations (F/Fmax) (XLS 5732 kb)

Muscle power (W/kg) (XLS 5742 kb)

Muscle length changes (m) (XLS 7645 kb)

Muscle shortening velocities (m/s) (XLS 7645 kb)
