# Supplementary material for: A real-time system for biomechanical analysis of human movement and muscle function
Source: Med Biol Eng Comput. 2013 Jul 25;51(10):1069–77. doi: 10.1007/s11517-013-1076-z (PMC3751375; doi:10.1007/s11517-013-1076-z)
Supplement: Supplementary file 1 — Detailed description of the model (PDF 672 kb) [file 11517_2013_1076_MOESM1_ESM.pdf]

SUPPLEMENTARY MATERIAL

This material is provided with the article: "A real-time system for biomechanical analysis of human movement and muscle function", by A.J. van den Bogert, T. Geijtenbeek, O. Even-Zohar, F. Steenbrink, and E.C. Hardin, published in Medical and Biological Engineering and Computing.

Marker set

The standard marker set for the Human Body Model (HBM) consists of 47 markers (Table 1, Figure 1). Some of the markers are required for model initialization, and must be placed at well-defined anatomical landmarks. Their coordinates must be available during model initialization. These markers are also used for the inverse kinematic analysis (equation 1 in main paper). The other markers are only used for the inverse kinematic analysis. These do not need to be accurately placed, and when missing, the movement analysis in HBM will still work until too many are missing and the Jacobian matrix of the least squares problem (equation 1 in main paper) becomes singular. Additional markers in this category may be added by the user for increased robustness and accuracy.

Model initialization

To initialize the model, the subject is placed in an initialization pose. Specifically, the subject is instructed to stand straight and symmetrically, with feet pointing forward. Arms are raised to be horizontal, and oriented with palms down and elbow flexion axis vertical. The latter can be verified by asking the subject to flex their elbows. The forearm must remain in the horizontal

plane. The arms are then lowered to about 45 degrees (Figure 1) and the 3D marker coordinates are captured. The subject is now free to move. Immediately after the data capture, the model is generated and the real-time data processing is started. In the following section, we describe how the model is generated from the standing data.

Joint centers

For the hip joint centers (RHJC and LHJC), we use the method by Bell et al. (1990) to define the joint center relative to the GTRO and ASIS markers on each side.

The knee joint centers are assumed to lie at the midpoint between the epicondyles. We wish to avoid using markers on the medial side of the knee, so we ask the user to provide the knee width of the subject and the marker diameter (Figure 2 in main paper, window on lower left).

The ankle joint centers are defined similarly based on the lateral malleolus markers.

The subtalar joint centers are placed 12 mm below the ankle joint (van den Bogert et al., 1994).

Toe joint centers are placed 10 mm below and 30 mm posterior from the toe marker. The toe joint center is not a joint for which kinematic and kinetic analysis is performed. Its only purpose is to help establish the foot reference frame.

The shoulder joint centers (RSJC and LSJC) are placed 35 mm below the acromion markers (de Leva, 1996).

The S1/L5 joint center (where rotation between pelvis and lumbar spine occurs) is placed relative to the pelvis markers according to Reynolds (1982).

A joint between lumbar spine and thorax is placed at the level of the xyphoid marker (de Leva, 1996), on the line that connects S1/L5 to the midpoint between the shoulder joint centers.

A neck joint is placed at the level of the chin (de Leva, 1996), which we assume to be 2 cm above the C7 marker, and on the line that connects S1/L5 to the midpoint between the shoulder joint centers.

Elbow joint centers are placed at the midpoint between medial and lateral epicondyles.

Wrist joint centers are placed at the midpoint between medial and lateral wrist markers.

Segment reference frames

Pelvis: Origin is midpoint between hip joint centers. Y is parallel to the line from right hip joint center to left hip joint center. Z is parallel to the line from S1/L5 to the midpoint between shoulder joint centers. X is the cross product of Y and Z. Y is recalculated as the cross product of Z and X to ensure orthogonal axes.

Midtrunk: origin is S1/L5. XYZ axes are parallel to the Pelvis XYZ axes during initialization.

Thorax: origin is the thoracolumbar joint center. Y is parallel to the line from right shoulder joint center to left shoulder joint center. Z is parallel to the line from S1/L5 to the midpoint between shoulder joint centers. X is the cross product of Y and Z. Y is recalculated as the cross product of Z and X to ensure orthogonal axes.

Head: origin is the neck joint center. Z is the line from origin to the THEAD marker. Y is the line from RHEAD to LHEAD marker. X is the cross product of Y and Z. Y is recalculated as the cross product of Z and X to ensure orthogonal axes.

Upper arm: Origin is the shoulder joint center. Z is the line from elbow joint center to shoulder joint center. X points anteriorly, lies in the global sagittal plane and is perpendicular to Z. Y is the cross product of Z and X.

Forearm: Origin is the elbow joint center. Z is the line from wrist joint center to elbow joint center. X points anteriorly, lies in the global sagittal plane and is perpendicular to Z. Y is the cross product of Z and X.

Hand: Origin is the wrist joint center. XYZ are parallel to the XYZ axes of the same-side forearm during standing.

Thigh: Origin is the hip joint center. Z is the line from knee joint center to hip joint center. X points anteriorly, lies in the global sagittal plane, and is perpendicular to Z. Y is the cross product of Z and X.

Shank: Origin is the knee joint center. Z is the line from ankle joint center to knee joint center. X points anteriorly, lies in the global sagittal plane, and is perpendicular to Z. Y is the cross product of Z and X.

Foot: Origin is the subtalar joint center. Z is the line from toe joint center to subtalar joint center. X points superiorly, lies in the global sagittal plane, and is perpendicular to Z. Y is the cross product of Z and X.

Inertial properties

Inertial properties are generated from total body mass and body segment lengths, using gender-specific regression models from De Leva (1996). Segment lengths are determined during initialization, from the segment origins as described above. Total body mass and gender are provided by the user.

Segment motion

The kinematic degrees of freedom in the model are defined using a hierarchical arrangement of body segments (Table 2). The individual generalized coordinates (Table 3) will now be described in detail.

The Pelvis has 6 kinematic degrees of freedom, relative to the global reference frame. The generalized coordiantes are the global coordinates of the pelvis origin, and three rotations in ZYX (yaw, pitch, roll) sequence.

There are three rotational degrees of freedom between Thorax and Pelvis. The rotations are equally distributed between the Pelvis-Midtrunk joint (i.e. S1L5) and the Midtrunk-Thorax joint (i.e. L1T12). At each joint, the rotation is carried out in a YXZ sequence (flexion, side bend, twist).

The head has three rotational degrees of freedom relative to the Thorax, and is described by the same YXZ sequence (flexion, side bend, twist) that is used in the trunk.

Each upper arm has six degrees of freedom relative to the thorax. Three generalized coordinates describe the 3D position of the upper arm origin relative to the thorax. The orientation of the upper arm relative to the thorax is described using helical angles (Woltring, 1994) to avoid gimbal lock in the kinematic analysis. The XYZ components of the helical angle vector are labeled, respectively, as: ab-adduction, flexion-extension, and internal-external rotation.

Each forearm segment has two rotational degrees of freedom relative to the corresponding upper arm, in the order of flexion (about the Y axis of the upper arm) and pronation (about the Z axis of the forearm).

Each hand segment has two rotational degrees of freedom relative to the corresponding forearm, in the order of flexion (about the X axis of the forearm) and abduction (about the Y axis of the hand).

Each femur segment has three rotational degrees of freedom relative to the pelvis. The rotations are carried out in the order of YXZ (flexion-abduction-rotation) as recommended by the ISB (Wu et al., 2002).

Each shank segment has one rotational degree of freedom relative to the corresponding femur segment. The rotation (flexion) is carried out about the Y axis of the femur.

Each foot segment has two rotational degrees of freedom relative to the corresponding shank segment (van den Bogert et al., 1994). The first rotation (plantarflexion) is carried out about the Y axis of the shank and located at the midpoint between the malleoli. The second rotation (pronation) is carried out about the subtalar joint axis, located 12 mm below the first axis (van den Bogert, 1994) and oriented according to the model of Isman and Inman (1969).

Most joints in the model have fewer than six degrees of freedom, which increases the robustness of the kinematic analysis and this is also required to obtain joint moments that are representative of muscle function. During initialization, however, each joint was considered to have six degrees of freedom. Those degrees of freedom that were eliminated in the kinematic analysis, were calculated once during initialization and then kept constant during the movement. The resulting model is subject-specific in its bone lengths and joint alignments. For example, for a subject with a valgus aligned knee, the model will have the same valgus alignment.

Muscles

The sources for the muscle models are described in the main paper. The coupling between the 300 muscle elements and the 44 skeleton degrees of freedom can be illustrated by the sparsity pattern of the moment arm matrix, shown in Figure 2. For formatting reasons, Figure 2 shows the transpose of the moment arm matrix **D** that was define in the main paper.

Downloadable data files

Excel files are provided with full HBM results for all 12 subjects, as well as the mean and average of all subjects. All data were ensemble averaged and provided as 100 samples, representing 0% to 99% of the gait cycle, in steps of 1%. Where appropriate, the results were normalized to body mass of the subject. The files are:

- subjects.xls – Subject characteristics.
- grf.xls – Ground reaction force variables for each foot: 3D force (N/kg), center of pressure (m), and free vertical moment (Nm/kg).
- COM.xls – 3D coordinates of the whole-body center of mass (m).
- dof.xls – Kinematic analysis results (meters and degrees)
- jointmoment.xls – Inverse dynamic analysis results (N/kg and Nm/kg)
- jointpower.xls – Joint power for each kinematic degree of freedom (W/kg)
- loads.xls – 6-DOF intersegmental loads (N/kg and Nm/kg)
- muscleforce.xls – Muscle forces (N/kg)
- muscleactivation.xls – Muscle activations (F/Fmax)
- musclepower.xls – Muscle power (W/kg)
- musclelength.xls – Muscle length changes (m)

- musclevelocity.xls – Muscle shortening velocities (m/s)

The data may be considered representative for our subject population and test protocol. For other populations or test protocols, caution is advised when comparing results from other studies to these data.

References

Bell AL, Pedersen DR, Brand RA (1990) A comparison of the accuracy of several hip center location prediction methods. J Biomech 23: 617-621.

de Leva P (1996) Adjustments to Zatsiorsky-Seluyanov's segment inertia parameters. J Biomech 29: 1223-1230.

Isman RE, Inman VT (1969) Anthropometric studies of the human foot an ankle. Bull Prosth Res 11: 97-129.

Reynolds HM, Snow CC, Young JW (1982) Spatial geometry of the human pelvis. Oklahoma City: Technical Report FFA aeromedical institute. Cited by J. van Dieen (personal communication, May 21, 2010).

van den Bogert AJ, Smith GD, Nigg BM (1994) In vivo determination of the anatomical axes of the ankle joint complex: an optimization approach. J Biomech 27: 1477-1488.

Woltring HJ (1994) 3-D attitude representation of human joints: a standardization proposal. J Biomech 27: 1399-1414.

Wu G, Siegler S, Allard P, Kirtley C, Leardini A, Rosenbaum D, Whittle M, D'Lima DD, Cristofolini L, Witte H, Schmid O, Stokes I (2002) ISB recommendation on definitions of joint coordinate system of various joints for the reporting of human joint motion--part I: ankle, hip, and spine. International Society of Biomechanics. ; Standardization and Terminology Committee of the International Society of Biomechanics. J Biomech 35 : 543-548.

Table 1: The 47 markers used in the Human Body Model.

Markers required for model initialization		Additional markers used for inverse kinematics	
LASIS	Left anterior superior iliac spine	SACR	Sacrum
RASIS	Right anterior superior iliac spine	T10	10th Thoracic Vertebra
LPSIS	Left posterior superior iliac spine	NAVE	Navel
RPSIS	Right posterior superior iliac spine	STRN	Sternum
LGTRO	Right greater trochanter	LDELT	Left deltoid
RGTRO	Left greater trochanter	RDELT	Right deltoid
LLEK	Left lateral epicondyle of knee	RFIN	Right 3rd MCP joint
RLKE	Right lateral epicondyle of knee	LFIN	Left 3rd MCP joint
LLM	Left lateral malleolus	BBAC	Back thorax
RLM	Right lateral malleolus	FRTHI	Right front thigh
RTOE	Left second toe	FLTHI	Left front thigh
LTOE	Right second toe	RATI	Right anterior tibia
LSHO	Left acromion	LATI	Left anterior tibia
RSHO	Right acromion	RHEE	Right heel
LLEE	Left lateral epicondyle of the elbow	LHEE	Left heel
RLEE	Right lateral epicondyle of the elbow	RMT5	Right 5th MTP joint
LMEE	Left medial epicondyle of elbow	LMT5	Left 5th MTP joint
RMEE	Right medial epicondyle of elbow	RFRM	Right forearm
LLW	Left lateral wrist	LFRM	Left forearm
RLW	Right lateral wrist	FHEAD	Front of head
LMW	Left lateral wrist		
RMW	Right lateral wrist		
XYPH	Xyphoid process		
C7	7th cervical vertebra		
THEAD	Top of head		
RHEAD	Right side of head		
LHEAD	Left side of head		

Table 2: Hierarchical structure and degrees of freedom of the kinematic model. The two spine joints are kinematically coupled and share three rotational degrees of freedom.

<table>
<tr><th>Body segment</th><th>Parent segment</th><th>Number of degrees of freedom</th></tr>
<tr><td>Pelvis</td><td>Ground</td><td>6</td></tr>
<tr><td>MidTrunk</td><td>Pelvis</td><td rowspan="2">3</td></tr>
<tr><td>Thorax</td><td>MidTrunk</td></tr>
<tr><td>Head</td><td>Thorax</td><td>3</td></tr>
<tr><td>RUpperArm</td><td>Thorax</td><td>6</td></tr>
<tr><td>RForeArm</td><td>RUpperArm</td><td>2</td></tr>
<tr><td>RHand</td><td>RForeArm</td><td>2</td></tr>
<tr><td>LUpperArm</td><td>Thorax</td><td>6</td></tr>
<tr><td>LForeArm</td><td>LUpperArm</td><td>2</td></tr>
<tr><td>LHand</td><td>LForeArm</td><td>2</td></tr>
<tr><td>RThigh</td><td>Pelvis</td><td>3</td></tr>
<tr><td>RShank</td><td>RThigh</td><td>1</td></tr>
<tr><td>RFoot</td><td>RShank</td><td>2</td></tr>
<tr><td>LThigh</td><td>Pelvis</td><td>3</td></tr>
<tr><td>LShank</td><td>LThigh</td><td>1</td></tr>
<tr><td>LFoot</td><td>LShank</td><td>2</td></tr>
</table>

Table 3: Kinematic degrees of freedom in the HBM

Generalized variable	Name
q_1	PelvisX
q_2	PelvisY
q_3	PelvisZ
q_4	PelvisYaw
q_5	PelvisForwardPitch
q_6	PelvisRightRoll
q_7	TrunkFlexion
q_8	TrunkRightBend
q_9	TrunkLeftTwist
q_{10}	HeadFlexion
q_{11}	HeadRightBend
q_{12}	HeadLeftTwist
q_{13}	RShoulderUp
q_{14}	LShoulderUp
q_{15}	RShoulderForward
q_{16}	LShoulderForward
q_{17}	RShoulderInward
q_{18}	LShoulderInward
q_{19}	RShoulderFlexion
q_{20}	LShoulderFlexion
q_{21}	RShoulderAbduction
q_{22}	LShoulderAbduction
q_{23}	RShoulderInternalRotation
q_{24}	LShoulderInternalRotation
q_{25}	RElbowFlexion
q_{26}	LElbowFlexion
q_{27}	RForeArmPronation
q_{28}	LForeArmPronation
q_{29}	RWristFlexion
q_{30}	LWristFlexion

q_{31}	RHandAbduction
q_{32}	LHandAbduction
q_{33}	RHipFlexion
q_{34}	LHipFlexion
q_{35}	RHipAbduction
q_{36}	LHipAbduction
q_{37}	RHipInternalRotation
q_{38}	LHipInternalRotation
q_{39}	RKneeFlexion
q_{40}	LKneeFlexion
q_{41}	RAnklePlantarFlexion
q_{42}	LAnklePlantarFlexion
q_{43}	RFootPronation
q_{44}	LFootPronation

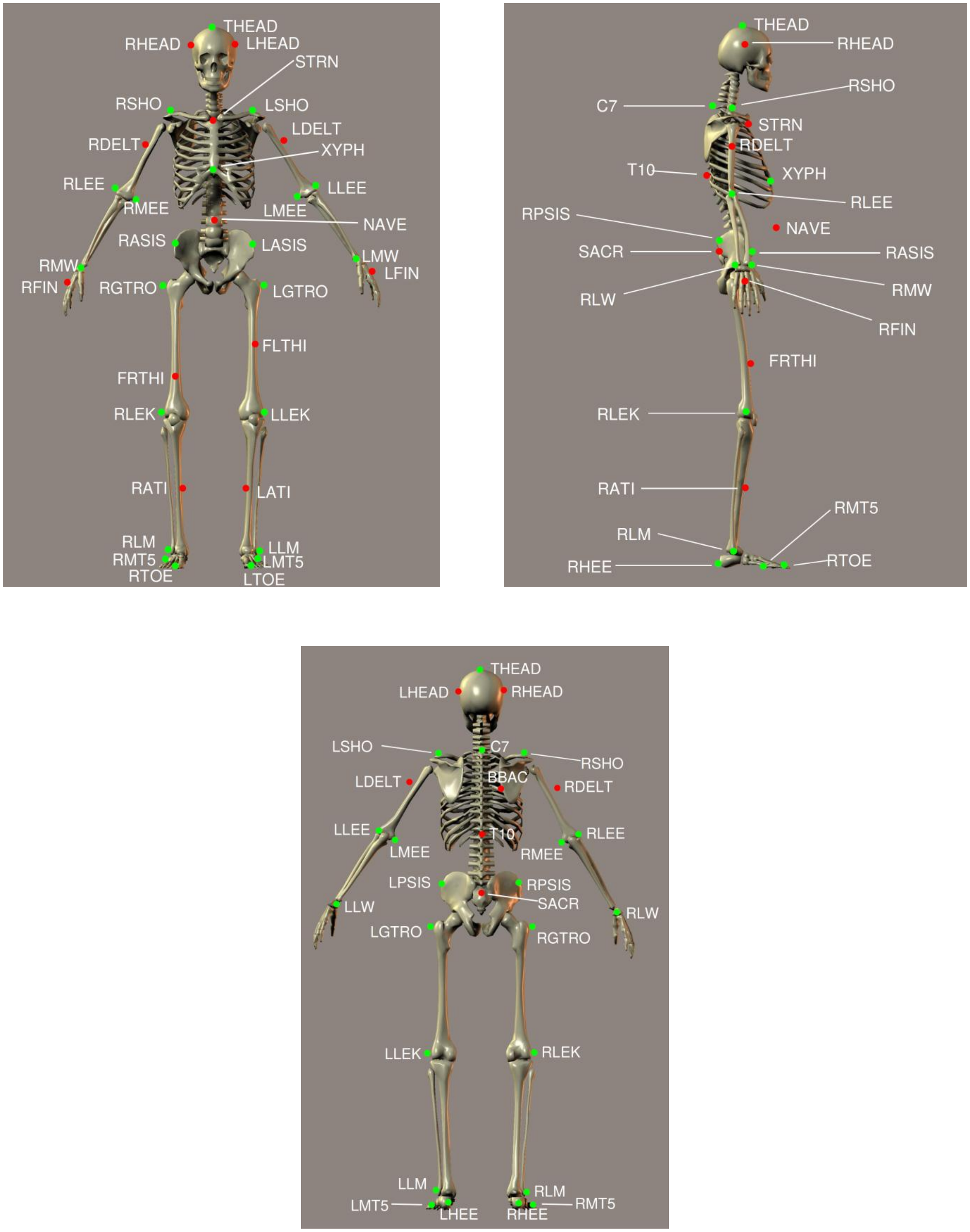

Figure 1: Marker set for the Human Body Model (HBM), shown from front, right side, and back. See text for details.

	PelvisX	PelvisY	PelvisZ	PelvisYaw	PelvisForwardPitch	PelvisRightRoll	TrunkFlexion	TrunkRightBend	TrunkLeftTwist	HeadFlexion	HeadRightBend	HeadLeftTwist	RShoulderUp	LShoulderUp	RShoulderForward	LShoulderForward	RShoulderInward	LShoulderInward	RShoulderFlexion	LShoulderFlexion	RShoulderAbduction	LShoulderAbduction	RShoulderInternalRotation	LShoulderInternalRotation	RElbowFlexion	LElbowFlexion	RForeArmPronation	LForeArmPronation	RWristFlexion	LWristFlexion	RHandAbduction	LHandAbduction	RHipFlexion	LHipFlexion	RHipAbduction	LHipAbduction	RHipInternalRotation	LHipInternalRotation	RKneeFlexion	LKneeFlexion	RAnklePlantarFlexion	LAnklePlantarFlexion	RFootPronation	LFootPronation
R GluteusMedius1																																	•		•		•							
R GluteusMedius2																																	•		•		•							
R GluteusMedius3																																	•		•		•							
R GluteusMinimus1																																	•		•		•							
R GluteusMinimus2																																	•		•		•							
R GluteusMinimus3																																	•		•		•							
R Semimembranosus																																	•		•				•					
R Semitendinosus																																	•		•		•		•					
R BicepsFemorisLH																																	•		•		•		•					
R Sartorius																																	•		•		•		•					
R AdductorLongus																																	•		•		•							
R AdductorBrevis																																	•		•		•							
R AdductorMagnus1																																	•		•		•							
R AdductorMagnus2																																	•		•		•							
R AdductorMagnus3																																	•		•		•							
R TensorFasciaLata																																	•		•		•		•					
R Pectineus																																	•		•		•							
R Gracilis																																	•		•		•		•					
R GluteusMaximus1																																	•		•		•							
R GluteusMaximus2																																	•		•		•							
R GluteusMaximus3																																	•		•		•							
R Iliacus																																	•		•		•							
R Psoas							•	•	•																								•		•		•							
R QuadratusFemoris																																	•		•		•							
R Gemelli																																	•		•		•							
R Piriformis																																	•		•		•							
R RectusFemoris																																	•		•		•		•					
R BicepsFemorisSH																																							•					
R VastusMedialis																																							•					
R VastusIntermedius																																							•					
R VastusLateralis																																							•					
R MedialGastroc																																							•		•		•	
R LateralGastroc																																							•		•		•	
R Soleus																																									•		•	
R TibialisPosterior																																									•		•	
R FlexDigLongus																																									•		•	
R FlexHalLongus																																									•		•	
R TibialisAnterior																																									•		•	
R PeroneusBrevis																																									•		•	
R PeroneusLongus																																									•		•	
R PeroneusTertius																																									•		•	
R ExtensorDigLongus																																									•		•	
R ExtensorHalLongus																																									•		•	
L GluteusMedius1																																		•		•		•						
L GluteusMedius2																																		•		•		•						
L GluteusMedius3																																		•		•		•						
L GluteusMinimus1																																		•		•		•						
L GluteusMinimus2																																		•		•		•						
L GluteusMinimus3																																		•		•		•						
L Semimembranosus																																		•		•				•				
L Semitendinosus																																		•		•		•		•				
L BicepsFemorisLH																																		•		•		•		•				
L Sartorius																																		•		•		•		•				
L AdductorLongus																																		•		•		•						
L AdductorBrevis																																		•		•		•						
L AdductorMagnus1																																		•		•		•						
L AdductorMagnus2																																		•		•		•						
L AdductorMagnus3																																		•		•		•						
L TensorFasciaLata																																		•		•		•		•				
L Pectineus																																		•		•		•						

Figure 2a: Coupling between skeleton degrees of freedom (44 columns) and muscle elements (300 rows). Muscle elements 1-60 are shown here.

	PelvisX	PelvisY	PelvisZ	PelvisYaw	PelvisForwardPitch	PelvisRightRoll	TrunkFlexion	TrunkRightBend	TrunkLeftTwist	HeadFlexion	HeadRightBend	HeadLeftTwist	RShoulderUp	LShoulderUp	RShoulderForward	LShoulderForward	RShoulderInward	LShoulderInward	RShoulderFlexion	LShoulderFlexion	RShoulderAbduction	LShoulderAbduction	RShoulderInternalRotation	LShoulderInternalRotation	RElbowFlexion	LElbowFlexion	RForeArmPronation	LForeArmPronation	RWristFlexion	LWristFlexion	RHandAbduction	LHandAbduction	RHipFlexion	LHipFlexion	RHipAbduction	LHipAbduction	RHipInternalRotation	LHipInternalRotation	RKneeFlexion	LKneeFlexion	RAnklePlantarFlexion	LAnklePlantarFlexion	RFootPronation	LFootPronation
L Gracilis																																		●		●		●		●				
L GluteusMaximus1																																		●		●		●						
L GluteusMaximus2																																		●		●		●						
L GluteusMaximus3																																		●		●		●						
L Iliacus																																		●		●		●						
L Psoas							●	●	●																									●		●		●						
L QuadratusFemoris																																		●		●		●						
L Gemelli																																		●		●		●						
L Piriformis																																		●		●		●						
L RectusFemoris																																		●		●		●		●				
L BicepsFemorisSH																																								●				
L VastusMedialis																																								●				
L VastusIntermedius																																								●				
L VastusLateralis																																								●				
L MedialGastroc																																								●		●		●
L LateralGastroc																																								●		●		●
L Soleus																																										●		●
L TibialisPosterior																																										●		●
L FlexDigLongus																																										●		●
L FlexHalLongus																																										●		●
L TibialisAnterior																																										●		●
L PeroneusBrevis																																										●		●
L PeroneusLongus																																										●		●
L PeroneusTertius																																										●		●
L ExtensorDigLongus																																										●		●
L ExtensorHalLongus																																										●		●
R DeltoidScapular1																			●		●		●																					
R DeltoidScapular2																			●		●		●																					
R DeltoidScapular3																			●		●		●																					
R DeltoidScapular4																			●		●		●																					
R DeltoidScapular5																			●		●		●																					
R DeltoidScapular6																			●		●		●																					
R DeltoidScapular7																			●		●		●																					
R DeltoidScapular8																			●		●		●																					
R DeltoidScapular9																			●		●		●																					
R DeltoidScapular10																			●		●		●																					
R DeltoidScapular11																			●		●		●																					
R DeltoidClavicular1																			●		●		●																					
R DeltoidClavicular2																			●		●		●																					
R DeltoidClavicular3																			●		●		●																					
R DeltoidClavicular4																			●		●		●																					
R CoracoBrachialis1																			●		●		●																					
R CoracoBrachialis2																			●		●		●																					
R CoracoBrachialis3																			●		●		●																					
R Infraspinatus1																			●		●		●																					
R Infraspinatus2																			●		●		●																					
R Infraspinatus3																			●		●		●																					
R Infraspinatus4																			●		●		●																					
R Infraspinatus5																			●		●		●																					
R Infraspinatus6																			●		●		●																					
R TeresMinor1																			●		●		●																					
R TeresMinor2																			●		●		●																					
R TeresMinor3																			●		●		●																					
R TeresMajor1																			●		●		●																					
R TeresMajor2																			●		●		●																					
R TeresMajor3																			●		●		●																					
R TeresMajor4																			●		●		●																					
R Supraspinatus1																			●		●		●																					
R Supraspinatus2																			●		●		●																					
R Supraspinatus3																			●		●		●																					

Figure 2b: Coupling between skeleton degrees of freedom (44 columns) and muscle elements (300 rows). Muscle elements 61-120 are shown here.

	PelvisX	PelvisY	PelvisZ	PelvisYaw	PelvisForwardPitch	PelvisRightRoll	TrunkFlexion	TrunkRightBend	TrunkLeftTwist	HeadFlexion	HeadRightBend	HeadLeftTwist	RShoulderUp	LShoulderUp	RShoulderForward	LShoulderForward	RShoulderInward	LShoulderInward	RShoulderFlexion	LShoulderFlexion	RShoulderAbduction	LShoulderAbduction	RShoulderInternalRotation	LShoulderInternalRotation	RElbowFlexion	LElbowFlexion	RForeArmPronation	LForeArmPronation	RWristFlexion	LWristFlexion	RHandAbduction	LHandAbduction	RHipFlexion	LHipFlexion	RHipAbduction	LHipAbduction	RHipInternalRotation	LHipInternalRotation	RKneeFlexion	LKneeFlexion	RAnklePlantarFlexion	LAnklePlantarFlexion	RFootPronation	LFootPronation
R Supraspinatus4																			•		•		•																					
R Subscapularis1																			•		•		•																					
R Subscapularis2																			•		•		•																					
R Subscapularis3																			•		•		•																					
R Subscapularis4																			•		•		•																					
R Subscapularis5																			•		•		•																					
R Subscapularis6																			•		•		•																					
R Subscapularis7																			•		•		•																					
R Subscapularis8																			•		•		•																					
R Subscapularis9																			•		•		•																					
R Subscapularis10																			•		•		•																					
R Subscapularis11																			•		•		•																					
R BicepsBrachiiLH																			•		•		•		•		•																	
R BicepsBrachiiSH1																			•		•		•		•		•																	
R BicepsBrachiiSH2																			•		•		•		•		•																	
R TricepsLH1																			•		•		•		•																			
R TricepsLH2																			•		•		•		•																			
R TricepsLH3																			•		•		•		•																			
R TricepsLH4																			•		•		•		•																			
R LatissimusDorsi1																			•		•		•																					
R LatissimusDorsi2																			•		•		•																					
R LatissimusDorsi3																			•		•		•																					
R LatissimusDorsi4																			•		•		•																					
R LatissimusDorsi5							•	•	•										•		•		•																					
R LatissimusDorsi6							•	•	•										•		•		•																					
R PectoralisMajorTH1																			•		•		•																					
R PectoralisMajorTH2																			•		•		•																					
R PectoralisMajorTH3																			•		•		•																					
R PectoralisMajorTH4																			•		•		•																					
R PectoralisMajorTH5																			•		•		•																					
R PectoralisMajorTH6																			•		•		•																					
R PectoralisMajorCH1																			•		•		•																					
R PectoralisMajorCH2																			•		•		•																					
R TricepsMedH1																									•																			
R TricepsMedH2																									•																			
R TricepsMedH3																									•																			
R TricepsMedH4																									•																			
R TricepsMedH5																									•																			
R Brachialis1																									•																			
R Brachialis2																									•																			
R Brachialis3																									•																			
R Brachialis4																									•																			
R Brachialis5																									•																			
R Brachialis6																									•																			
R Brachialis7																									•																			
R Brachioradialis1																									•																			
R Brachioradialis2																									•																			
R Brachioradialis3																									•																			
R PronatorTeres1																									•		•																	
R PronatorTeres2																											•																	
R Supinator1																											•																	
R Supinator2																											•																	
R Supinator3																											•																	
R Supinator4																											•																	
R Supinator5																											•																	
R PronatorQuadratus1																											•																	
R PronatorQuadratus2																											•																	
R PronatorQuadratus3																											•																	
R TricepsLatH1																									•																			
R TricepsLatH2																									•																			

Figure 2c: Coupling between skeleton degrees of freedom (44 columns) and muscle elements (300 rows). Muscle elements 121-180 are shown here.

	PelvisX	PelvisY	PelvisZ	PelvisYaw	PelvisForwardPitch	PelvisRightRoll	TrunkFlexion	TrunkRightBend	TrunkLeftTwist	HeadFlexion	HeadRightBend	HeadLeftTwist	RShoulderUp	LShoulderUp	RShoulderForward	LShoulderForward	RShoulderInward	LShoulderInward	RShoulderFlexion	LShoulderFlexion	RShoulderAbduction	LShoulderAbduction	RShoulderInternalRotation	LShoulderInternalRotation	RElbowFlexion	LElbowFlexion	RForeArmPronation	LForeArmPronation	RWristFlexion	LWristFlexion	RHandAbduction	LHandAbduction	RHipFlexion	LHipFlexion	RHipAbduction	LHipAbduction	RHipInternalRotation	LHipInternalRotation	RKneeFlexion	LKneeFlexion	RAnklePlantarFlexion	LAnklePlantarFlexion	RFootPronation	LFootPronation
R_TricepsLatH3																								•																			
R_TricepsLatH4																								•																			
R_TricepsLatH5																								•																			
R_Anconeus1																								•																			
R_Anconeus2																								•																			
R_Anconeus3																								•																			
R_Anconeus4																								•																			
R_Anconeus5																								•																			
L_DeltoidScapular1																			•		•		•																				
L_DeltoidScapular2																			•		•		•																				
L_DeltoidScapular3																			•		•		•																				
L_DeltoidScapular4																			•		•		•																				
L_DeltoidScapular5																			•		•		•																				
L_DeltoidScapular6																			•		•		•																				
L_DeltoidScapular7																			•		•		•																				
L_DeltoidScapular8																			•		•		•																				
L_DeltoidScapular9																			•		•		•																				
L_DeltoidScapular10																			•		•		•																				
L_DeltoidScapular11																			•		•		•																				
L_DeltoidClavicular1																			•		•		•																				
L_DeltoidClavicular2																			•		•		•																				
L_DeltoidClavicular3																			•		•		•																				
L_DeltoidClavicular4																			•		•		•																				
L_CoracoBrachialis1																			•		•		•																				
L_CoracoBrachialis2																			•		•		•																				
L_CoracoBrachialis3																			•		•		•																				
L_Infraspinatus1																			•		•		•																				
L_Infraspinatus2																			•		•		•																				
L_Infraspinatus3																			•		•		•																				
L_Infraspinatus4																			•		•		•																				
L_Infraspinatus5																			•		•		•																				
L_Infraspinatus6																			•		•		•																				
L_TeresMinor1																			•		•		•																				
L_TeresMinor2																			•		•		•																				
L_TeresMinor3																			•		•		•																				
L_TeresMajor1																			•		•		•																				
L_TeresMajor2																			•		•		•																				
L_TeresMajor3																			•		•		•																				
L_TeresMajor4																			•		•		•																				
L_Supraspinatus1																			•		•		•																				
L_Supraspinatus2																			•		•		•																				
L_Supraspinatus3																			•		•		•																				
L_Supraspinatus4																			•		•		•																				
L_Subscapularis1																			•		•		•																				
L_Subscapularis2																			•		•		•																				
L_Subscapularis3																			•		•		•																				
L_Subscapularis4																			•		•		•																				
L_Subscapularis5																			•		•		•																				
L_Subscapularis6																			•		•		•																				
L_Subscapularis7																			•		•		•																				
L_Subscapularis8																			•		•		•																				
L_Subscapularis9																			•		•		•																				
L_Subscapularis10																			•		•		•																				
L_Subscapularis11																			•		•		•																				
L_BicepsBrachiiLH																			•		•		•		•		•																
L_BicepsBrachiiSH1																			•		•		•		•		•																
L_BicepsBrachiiSH2																			•		•		•		•		•																
L_TricepsLH1																			•		•		•		•																		
L_TricepsLH2																			•		•		•		•																		
L_TricepsLH3																			•		•		•		•																		

Figure 2d: Coupling between skeleton degrees of freedom (44 columns) and muscle elements (300 rows). Muscle elements 181-240 are shown here.

	PelvisX	PelvisY	PelvisZ	PelvisYaw	PelvisForwardPitch	PelvisRightRoll	TrunkFlexion	TrunkRightBend	TrunkLeftTwist	HeadFlexion	HeadRightBend	HeadLeftTwist	RShoulderUp	LShoulderUp	RShoulderForward	LShoulderForward	RShoulderInward	LShoulderInward	RShoulderFlexion	LShoulderFlexion	RShoulderAbduction	LShoulderAbduction	RShoulderInternalRotation	LShoulderInternalRotation	RElbowFlexion	LElbowFlexion	RForeArmPronation	LForeArmPronation	RWristFlexion	LWristFlexion	RHandAbduction	LHandAbduction	RHipFlexion	LHipFlexion	RHipAbduction	LHipAbduction	RHipInternalRotation	LHipInternalRotation	RKneeFlexion	LKneeFlexion	RAnklePlantarFlexion	LAnklePlantarFlexion	RFootPronation	LFootPronation
L TricepsLH4																				•		•		•		•																		
L LatissimusDorsi1																				•		•		•																				
L LatissimusDorsi2																				•		•		•																				
L LatissimusDorsi3																				•		•		•																				
L LatissimusDorsi4																				•		•		•																				
L LatissimusDorsi5							•	•	•											•		•		•																				
L LatissimusDorsi6							•	•	•											•		•		•																				
L PectoralisMajorTH1																				•		•		•																				
L PectoralisMajorTH2																				•		•		•																				
L PectoralisMajorTH3																				•		•		•																				
L PectoralisMajorTH4																				•		•		•																				
L PectoralisMajorTH5																				•		•		•																				
L PectoralisMajorTH6																				•		•		•																				
L PectoralisMajorCH1																				•		•		•																				
L PectoralisMajorCH2																				•		•		•																				
L TricepsMedH1																										•																		
L TricepsMedH2																										•																		
L TricepsMedH3																										•																		
L TricepsMedH4																										•																		
L TricepsMedH5																										•																		
L Brachialis1																										•																		
L Brachialis2																										•																		
L Brachialis3																										•																		
L Brachialis4																										•																		
L Brachialis5																										•																		
L Brachialis6																										•																		
L Brachialis7																										•																		
L Brachioradialis1																										•																		
L Brachioradialis2																										•																		
L Brachioradialis3																										•																		
L PronatorTeres1																										•		•																
L PronatorTeres2																												•																
L Supinator1																												•																
L Supinator2																												•																
L Supinator3																												•																
L Supinator4																												•																
L Supinator5																												•																
L PronatorQuadratus1																												•																
L PronatorQuadratus2																												•																
L PronatorQuadratus3																												•																
L TricepsLatH1																										•																		
L TricepsLatH2																										•																		
L TricepsLatH3																										•																		
L TricepsLatH4																										•																		
L TricepsLatH5																										•																		
L Anconeus1																										•																		
L Anconeus2																										•																		
L Anconeus3																										•																		
L Anconeus4																										•																		
L Anconeus5																										•																		
L ErectorSpinae							•	•	•																																			
R ErectorSpinae							•	•	•																																			
L ObliqueExternal							•	•	•																																			
R ObliqueExternal							•	•	•																																			
L ObliqueInternal							•	•	•																																			
R ObliqueInternal							•	•	•																																			
L QuadratusLumborum							•	•	•																																			
R QuadratusLumborum							•	•	•																																			
L RectusAbdominis							•	•	•																																			
R RectusAbdominis							•	•	•																																			

Figure 2e: Coupling between skeleton degrees of freedom (44 columns) and muscle elements (300 rows). Muscle elements 241-300 are shown here.
